# Regulatory mechanisms and function of hypoxia-induced long noncoding RNA *NDRG1-OT1* in breast cancer cells

**DOI:** 10.1038/s41419-022-05253-2

**Published:** 2022-09-20

**Authors:** Hsing-Hua Chao, Jun-Liang Luo, Ming-Hsuan Hsu, Li-Han Chen, Tzu-Pin Lu, Mong-Hsun Tsai, Eric Y. Chuang, Li-Ling Chuang, Liang-Chuan Lai

**Affiliations:** 1 Graduate Institute of Physiology, College of Medicine, National Taiwan University, Taipei, Taiwan; 2 Institute of Fisheries Science, College of Life Science, National Taiwan University, Taipei, Taiwan; 3 Institute of Epidemiology and Preventive Medicine, National Taiwan University, Taipei, Taiwan; 4 Bioinformatics and Biostatistics Core, Center of Genomic and Precision Medicine, National Taiwan University, Taipei, Taiwan; 5 Institute of Biotechnology, National Taiwan University, Taipei, Taiwan; 6 Graduate Institute of Biomedical Electronics and Bioinformatics, National Taiwan University, Taipei, Taiwan; 7 College of Biomedical Engineering, China Medical University, Taichung, Taiwan; 8 School of Physical Therapy and Graduate Institute of Rehabilitation Science, College of Medicine, Chang Gung University, Taoyuan, Taiwan; 9 Department of Physical Medicine and Rehabilitation, Chang Gung Memorial Hospital at Linkou, Taoyuan, Taiwan

**Keywords:** Breast cancer, Long non-coding RNAs

## Abstract

Hypoxia is a classic feature of the tumor microenvironment that has profound effects on cancer progression and is tightly associated with poor prognosis. Long noncoding RNAs (lncRNAs), a component of the noncoding genome, have been increasingly investigated due to their diverse roles in tumorigenesis. Previously, a hypoxia-induced lncRNA, *NDRG1-OT1*, was identified in MCF-7 breast cancer cells using next-generation sequencing. However, the regulatory mechanisms of *NDRG1-OT1* remain elusive. Therefore, the purpose of this study was to investigate the regulatory mechanisms and functional roles of *NDRG1-OT1* in breast cancer cells. Expression profiling of *NDRG1-OT1* revealed that it was upregulated under hypoxia in different breast cancer cells. Overexpression and knockdown of HIF-1α up- and downregulated *NDRG1-OT1*, respectively. Luciferase reporter assays and chromatin immunoprecipitation assays validated that HIF-1α transcriptionally activated *NDRG1-OT1* by binding to its promoter (−1773 to −1769 and −647 to −643 bp). Next, to investigate whether *NDRG1-OT1* could function as a miRNA sponge, results of in silico analysis, expression profiling of predicted miRNAs, and RNA immunoprecipitation assays indicated that *NDRG1-OT1* could act as a miRNA sponge of *miR-875-3p*. In vitro and in vivo functional assays showed that *NDRG1-OT1* could promote tumor growth and migration. Lastly, a small peptide (66 a.a.) translated from *NDRG1-OT1* was identified. In summary, our findings revealed novel regulatory mechanisms of *NDRG1-OT1* by HIF-1α and upon *miR-875-3p*. Also, *NDRG1-OT1* promoted the malignancy of breast cancer cells and encoded a small peptide.

## Introduction

Breast cancer is the most commonly diagnosed cancer and a major cause of death in women worldwide [1, 2]. This disease is characterized by heterogeneous cell populations and causes great challenges in terms of therapeutic strategies [3]. Cancer has characteristics like unrestricted replication, altered energy metabolism, resistance to apoptosis, and hypoxia. Owing to insufficient angiogenesis in the developing lesion, cancer cells are subject to hypoxia due to imbalances in oxygen delivery and supply [4]. Hypoxia within the tumor microenvironment has been extensively studied for decades and affects malignant cells in various ways [5]. About 25–40% of invasive breast tumors exhibit hypoxic regions [6]. Although hypoxic conditions are detrimental to cancer cells, hypoxia imposes selective pressure that promotes adaptation and malignant progression [7, 8]. Therefore, the presence of hypoxic regions within solid tumors is a dependable indicator of poor prognosis, because hypoxia within solid tumors is typically associated with increased resistance to chemotherapy, immunotherapy, and radiation therapy [9].

In most solid tumors, adaptation to decreased O_2_ availability is achieved primarily through the activity of two hypoxia-inducible factors, HIF-1 and HIF-2 [10]. HIFs are basic helix-loop-helix DNA binding proteins that function as heterodimers, composed of an oxygen-regulated alpha subunit and a stably expressed beta subunit [11]. Under conditions of normal oxygen tension, the alpha subunit is hydroxylated at key proline residues, which inhibits its transactivation function and induces proteasomal degradation [12]. Whereas, under hypoxia conditions, HIF-1α translocates into the nucleus to activate hypoxia-responsive genes. Genes activated by HIF-1 function in apoptosis, angiogenesis, glycolysis cascades, remodeling of the extracellular matrix, and cell cycle control [13–15]. HIF-2 also plays a role in modulating a variety of widely expressed hypoxia-inducible genes [16]. These alterations induce the survival of malignant cells in the hypoxic microenvironment and lead to a worse prognosis. However, the detailed mechanisms of the links between hypoxia and tumor progression remain to be determined.

Current evidence suggests that at least 75% of the genome is transcribed into noncoding RNAs [17]. Among these noncoding transcripts, the ones that are longer than 200 nucleotides are classified as long noncoding RNAs (lncRNAs). Having characteristics such as low conservation, low expression levels, and diverse gene regulation mechanisms, lncRNAs have distinct features from other noncoding RNAs [18]. Regarding their roles in cancer, lncRNAs are differentially expressed in various tissues and participate in multiple stages of gene regulation to change cancer cell function [19]. For instance, *HOTAIR*, a polycomb-related lncRNA, enhances hypoxic cancer cell proliferation, migration, and invasion [20]. *UCA1*, a highly expressed lncRNA in bladder cancer, modulates the FGFR1/ERK pathway by acting as a microRNA (miRNA) sponge [21]. Definitive evidence for additional roles of lncRNAs in tumorigenesis is being worked out [22].

Numerous lncRNAs are subject to transcriptional regulation by factors that regulate different aspects of cellular homeostasis. For example, *CONCR*, a lncRNA transcriptionally activated by MYC, regulates sister chromatid cohesion by modulating DDX11 enzymatic activity [23]. *LincRNA-p21*, a lncRNA induced by p53, serves as a repressor in p53-dependent transcriptional responses [24]. *H19*, an oncogenic lncRNA in various cancers [25–27], is activated by HIF-1α through direct and indirect activity [28]. Beyond these observations, the transcriptional regulation of lncRNAs in breast cancer remains to be clarified.

*NDRG1-OT1* is one of the oxygen-responsive lncRNAs and is induced by hypoxia in breast cancer cells [29]. The gene locus of *NDRG1-OT1* overlaps that of *NDRG1*, a stress-responsive and highly conserved gene. *NDRG1-OT1*, like *NDRG1*, is upregulated in hypoxia, yet it promotes NDRG1 degradation via ubiquitin-mediated proteolysis in breast cancer cells [29]. In addition, different fragments of *NDRG1-OT1* have different effects on *NDRG1* transcription in MCF-7 breast cancer cells under hypoxia by recruiting distinct proteins [30]. Although *NDRG1-OT1* was found to be upregulated under hypoxia and downregulated after shifting to reoxygenation in MCF-7 cells [29], the regulatory mechanisms of *NDRG1-OT1* upon hypoxia have yet to be fully characterized. Therefore, the purpose of this study was to investigate whether HIFs could regulate *NDRG1-OT1* expression. Gain-of-function and loss-of-function assays of HIFs were performed. Luciferase reporter assays and chromatin immunoprecipitation (ChIP) assays verified the direct interaction between HIF-1α and the putative hypoxia response elements (HREs) in the promoter of *NDRG1-OT1*. In addition, miRNA expression and RNA immunoprecipitation suggested that *NDRG1-OT1* could serve as a miRNA sponge. Functional assays showed that *NDRG1-OT1* could promote the malignancy of triple-negative breast cancer cells and angiogenesis in peripheral endothelial cells. Lastly, western blotting and immunofluorescence staining revealed that the region between 29 and 226 bp of *NDRG1-OT1* encoded small peptides.

## Materials and methods

### Cell culture and treatments

MCF-7 and MDA-MB-231 breast cancer cells, human umbilical vein endothelial cells (HUVECs), and HEK293T human embryonic kidney cells were maintained in Dulbecco’s Modified Eagle Medium (DMEM) (GIBCO, Carlsbad, CA, USA). All were supplemented with 10% fetal bovine serum (FBS) (HyClone, Logan, UT, USA) and 1% antibiotics (penicillin-streptomycin) (GIBCO). The cell lines above were incubated at 37 °C in a humidified incubator at 5% CO_2_. Breast cancer MDA-MB-453 cells were cultured in Leibovitz’s L-15 (GIBCO) medium with 1% antibiotics (penicillin-streptomycin) and 10% FBS, and were at 37 °C in a humidified incubator at 0% CO_2_. In some experiments, cells were treated with 300 μM CoCl_2_ (Sigma, Saint Louis, MO, USA) to mimic hypoxic conditions or cultured in the hypoxia chamber (Ruskinn Technology, Bridgend, UK) filled with a gas mixture of 0.5% O_2_, 5% CO_2_, and 94.5% N_2_ for 24 h.

### Cell line authentication

Cell experiments were performed on cells that were passaged less than 20 times, and were routinely tested for mycoplasma using PCR Mycoplasma Detection Kit (ABM Inc., Vancouver, Canada). The identity of cell lines was authenticated by short-tandem repeat (STR) analysis (Mission Biotech Inc., Taipei, Taiwan).

### Plasmid construction

To overexpress *NDRG1-OT1* (https://lncipedia.org/db/transcript/lnc-NDRG1-1:4), construction of the expression plasmid pcDNA3.1(+)-*NDRG1-OT1* has been described previously [29]. To overexpress HIF-1α and HIF-2α under normoxic conditions, proline residues were changed to alanine to avoid hydroxylation. The pcDNA3-HIF-1α-P402A/P564A (Addgene plasmid # 18955), a HIF-1α mutant resistant to PHD2-mediated hydroxylation and VHL-mediated ubiquitination/degradation, and pcDNA3-HIF-2α-P405A/P531A (Addgene plasmid # 18956), a HIF-2α mutant resistant to PHD2-mediated hydroxylation and VHL-mediated ubiquitination/degradation, were purchased from Addgene (Addgene, Cambridge, MA, USA). To determine promoter activity by luciferase assay, the luciferase expression plasmid pGL3-*NDRG1-OT1* promoter was purchased from the BioMed Resource Core of the 1st Core Facility Lab, NTU-CM (Taipei, Taiwan). To create this plasmid, the *NDRG1-OT1* promoter region encompassing −2000 to −1 bp relative to the transcription start site (chr8:133,244,504-133,246,503) was amplified from human genomic DNA by PCR. The following primers with *Kpn*I and *Hind*III restriction sites were used: forward, 5’-TTTCTCTATCGATAGGTACCG AATTAATGATTTAATCTATCTGTTTATGAACATAT-3’; reverse, 5’-CCGGAATGCCAAGCTTTGCCTTACAAAGGAGGAACA -3’. The PCR product was subcloned into the *Kpn*I-*Hind*III sites of pGL3-basic vector, and the final construct was called pGL3-*NDRG1-OT1* promoter.

Two HIF-1α core binding motifs (HREs), located at −1773 to −1769 and −647 to −643 bp relative to the transcription start site of *NDRG1-OT1*, were predicted by RNAhybrid (https://bibiserv2.cebitec.uni-bielefeld.de/rnahybrid). Mutations in these HRE sequences of the pGL3-*NDRG1-OT1* promoter construct were generated using the Quick Change Lightning Site-Directed Mutagenesis Kit (Agilent Technologies, Santa Clara, CA, USA) according to the manufacturer’s instructions. The pairs of primers were designed with the website recommended by the manufacturer (www.agilent.com/genomics/qcpd) and are listed in Table S1. The newly constructed plasmids were transformed and extracted. In addition, the mutated sequences were validated by sequencing.

To verify the interaction between *NDRG1-OT1* and *miR-875-3p*, the plasmids pMIR-REPORT-*NDRG1-OT1*, pMIR-REPORT-*NDRG1-OT1* site 1 mutation, pMIR-REPORT-*NDRG1-OT1* site 2 mutation, and pMIR-REPORT-*NDRG1-OT1* site 1 + 2 mutation were constructed (BioMed Resource Core). The *NDRG1-OT1* 3’ UTR was inserted after luciferase (Ambion, Thermo Fisher, Waltham, MA, USA), and binding sites 1 (438–445 nt) or 2 (475–482 nt) or both were mutated by changing them to the complementary sequence. The two binding sites were predicted by miRDB (http://mirdb.org).

To identify whether *NDRG1-OT1* encodes translatable peptides, four regions of *NDRG1-OT1* containing putative peptide sequences were linked with FLAG epitope tags (GACTACAAGGACGACGATGACAAG) and inserted into the pcDNA3.1/ ZEO(+) vector (BioMed Resource Core). A start codon and an HA epitope tag (TACCCTTATGATGTGCCAGATTATGCC) were also inserted in front of *NDRG1-OT1* to serve as a positive control.

### Transfection, RNA interference, miRNA inhibitor

The *NDRG1-OT1* expression plasmid (pcDNA3.1(+)-*NDRG1-OT1*), HIF-1α expression plasmid (pcDNA3-HIF-1α-P402A/P564A), HIF-2α expression plasmid (pcDNA3-HIF-2α-P405A/P531A) and control plasmids (pcDNA3.1(+)) were transfected into cells using jetPRIME (Polyplus-transfection, Illkirch, France) reagent. *miR-875-3p* expression plasmid (pcDNA6.2-GW_EmGFP-*hsa-miR-875-3p*) and control plasmids (pcDNA6.2-GW_EmGFP) were transfected into cells using TransIT-X2 (Mirus Bio LLC, Madison, Wisconsin, USA) reagent according to the manufacturer’s instructions. For RNA interference, cells were transfected by a siGENOME-SMARTpool (GE Healthcare Dharmacon, Lafayette, CO, USA) consisting of four small interferings (si)RNAs (Table S2) targeting *HIF1A* or *HIF2A* using DharmaFECT 1 transfection reagent (GE Healthcare Dharmacon) according to the manufacturer’s protocol. The siGENOME Non-Targeting siRNA (Table S2) (GE Healthcare Dharmacon) was used as a negative control.

To examine the effect of *miR-875-3p* on *NDRG1-OT1*, MDA-MB-231 cells (1 × 10^6^ cells) were seeded into six-well plates, transfected with *miR-875-3p* expression plasmids, and treated with 50 nM anti-*miR-875-3p* inhibitor (Integrated DNA Technologies, Newark, NJ, USA). The cells were incubated for 24 h at 37 °C in 5% CO_2_.

### Antisense oligonucleotides against *NDRG1-OT1*

The Gapmer antisense oligonucleotides (ASO) against *NDRG1-OT1* was designed and synthesized by Genomics Inc (Taipei, Taiwan). The Gapmer ASOs were designed to have 14 nucleotides in length, with the first and last three bases having the locked nucleic acid (LNA) chemistry and phosphorothioate backbone modification. For transfection, *NDRG1-OT1*-targeting LNA gapmers at a final concentration of 1 nM were transfected with Lipofectamine® RNAiMAX (Life Technologies) according to the manufacturer’s instructions.

### Luciferase reporter assay

To determine the effect of HIF-1α on the HRE reporter construct and of *miR-875-3p* on the *NDRG1-OT1* 3’UTR, HEK293T cells were seeded in 24-well plates at a density of 8 × 10^4^ cells/well, and transfected 24 h later with 50 ng wild-type or mutant firefly luciferase reporter construct plus 5 ng Renilla luciferase plasmid (pGL4.74 [hRluc/TK], kindly provided by Dr. Meng-Chun Hu, NTU, Taipei) using jetPRIME (Polyplus-transfection) reagent. After 24 h, cells were cultured in fresh complete medium or medium with 300 μM CoCl_2_ and kept at 37 °C in a humidified incubator for 24 h. After cells were lysed in cell lysis buffer (92.8 mM K_2_HPO_4_, 9.2 mM KH_2_PO_4_, and 0.2% Triton X-100 in ddH_2_O), the luciferase activity was measured by using the Dual-Glo luciferase reporter assay system (Promega, Fitchburg, WI, USA) and normalized with respect to Renilla luciferase activity.

### RNA extraction, reverse transcription, and quantitative RT-PCR (qPCR)

Total RNA was isolated using NucleoZOL reagent (Machery-Nagel, Düren, Germany) according to the manufacturer’s instructions. One microgram of total RNA was reverse-transcribed by the High-Capacity cDNA Reverse Transcription Kit (Applied Biosystems, Carlsbad, CA, USA). For reverse transcription of miRNA, SuperScript IV Reverse Transcriptase (Invitrogen, Carlsbad, CA, USA) was used with the primers listed in Table S3. A total of 2.5% of each reaction was used as the template for quantitative RT-PCR (qPCR) with OmicsGreen qPCR MasterMix (OmicsBio, New Taipei City, Taiwan), and the reactions were performed on StepOnePlus Real-Time PCR System (Thermo Fisher, Waltham, MA, USA). The primer pairs used for the detection of cDNAs are listed in Table S3. Finally, the relative gene expression levels were normalized to 18 S rRNA, *GAPDH*, or *RNU44* using the 2^−ΔΔCt^ method.

### Nuclear-cytoplasmic RNA fractionation

To determine the subcellular localization of RNA, fractionation of nuclear and cytoplasmic RNA was performed by using the Cytoplasmic & Nuclear RNA Purification Kit (Norgen Biotek, Thorold, ON, Canada). Cells were first lysed with Lysis Buffer J (Norgen Biotek), and the lysate was separated through centrifugation, with the supernatant containing the cytoplasmic RNA and the pellet containing the nuclear RNA. Buffer SK (Norgen Biotek) and ethanol were then added to the cytoplasmic and nuclear fractions, and the solution was loaded onto a spin column to collect RNA. The bound RNA was then washed with Wash Solution A (Norgen Biotek) to remove the remaining impurities, and the purified RNA was eluted with Elution Buffer E (Norgen Biotek). The isolated RNA was subsequently reverse-transcribed, and the relative expression level was measured by qPCR. The pairs of primers used are listed in Table S3.

### Western blotting

Cells were lysed with RIPA Lysis Buffer (EMD Millipore, Billerica, MA, USA), and total protein concentrations were determined by Coomassie Protein Assay Reagent (Thermo Fisher). The extracted protein samples were separated by SDS-PAGE and transferred to PVDF membranes (GE healthcare, Chicago, IL, USA). The membranes were blocked with Lightning Blocking Buffer (Arrowtec Life Science, New Taipei City, Taiwan) and immunoblotted overnight at 4 °C with the following primary antibodies: HIF-1α (cat. no. GTX127309; GeneTex, Irvine, CA, USA; Proteintech, Chicago, IL, USA), GAPDH (cat. no. GTX100118; GeneTex), HIF-2α (cat. no. 26422-1-AP; Proteintech), NDRG1 (cat. no. ab124689; Abcam, Cambridge, England), FLAG (cat. no. AE063; BIOTOOLS, New Taipei City, Taiwan), HA (cat. no. 3724; Cell Signaling, Danvers, MA, USA). After hybridization, the membranes were washed with Tris-buffered saline with 0.1% Tween-20 (TBST) and incubated with horseradish peroxidase-conjugated anti-rabbit IgG (cat. no. GTX213110-01; GeneTex). The blotted proteins were detected using an enhanced chemiluminescence system (Millipore, Billerica, MA, USA) with the BioSpectrum Imaging System (UVP, Upland, CA, USA).

### Chromatin immunoprecipitation (ChIP)

To confirm the interaction between HIF-1α and the predicted HREs in *NDRG1-OT1*, ChIP was performed using the Pierce Magnetic ChIP Kit (Thermo Fisher). Cells were crosslinked with 1% formaldehyde in the medium for 10 min at room temperature, followed by neutralization with 125 mM glycine incubated at room temperature for 5 min. After two washing with cold phosphate-buffered saline, the cell pellets were lysed in Membrane Extraction Buffer (Thermo Fisher) and sheared chromatin was generated by incubation with micrococcal nuclease (MNase) (Thermo Fisher) in a 37 °C water bath for 15 min. The nuclei were sonicated with three 20 s pulses on ice to break the nuclear membrane, and the digested chromatin was incubated with the following primary antibodies: HIF-1α (cat. no. GTX127309; Genetex), p300 (cat. no. GTX30619; GeneTex), RNA polymerase II (cat. no. 49-1033; Thermo Fisher). Next, the DNA binding protein-DNA complexes were immunoprecipitated with ChIP Grade Protein A/G Magnetic Beads (Thermo Fisher). After washing the beads with IP wash buffers 1 and 2 (Thermo Fisher), the complexes were reverse-crosslinked by incubating at 65 °C for 40 min, followed by proteinase K treatment at 65 °C for 1.5 h to digest DNA binding proteins. The purified DNA was recovered in DNA Binding Buffer (Thermo Fisher), washed with DNA Column Wash Buffer (Thermo Fisher), and eluted with DNA Column Elution Solution (Thermo Fisher). The relative chromatin enrichment was measured by qPCR. The pairs of primers used are listed in Table S4.

### RNA immunoprecipitation (RIP)

To verify the interaction between *NDRG1-OT1* and Argonaute 2 (AGO2), the Magna RNA immunoprecipitation (RIP) Kit (Millipore) was used. MDA-MB-231 breast cancer cells (2 × 10^7^) were harvested with 1% trypsin-EDTA (GIBCO) and lysed with 100 μL RIP lysis buffer containing a proteinase inhibitor cocktail and RNase inhibitor (Millipore). The lysate was centrifuged at 14,000 rpm for 10 min. Then, 900 μL RIP immunoprecipitation buffer (Millipore) containing RIP wash buffer, 0.5% EDTA, and RNase inhibitor were added to the supernatant with 5 μg anti-AGO2 antibodies (cat. no. M00189; Boster Biological Technology, Pleasanton, CA, USA) or 5 μg anti-IgG control antibodies (cat. no. AQ127; Millipore) that were prebound on magnetic beads. The bead mixture was agitated overnight at 4 °C. Ten percent of the supernatant was saved as a record of the input. Beads were washed six times with RIP wash buffer and treated with proteinase K at 55 °C for 30 min. RNA was extracted using NucleoZOL reagent (Machery-Nagel) and reverse-transcribed, and the relative gene expression level was measured by qPCR. The pairs of primers used are listed in Table S3.

### MTT assay

MDA-MB-231 cells were seeded in 96-well plates at a density of 5000 cells/well and transfected 0.05 μg pcDNA3.1(+)-*NDRG1-OT1* or empty vector (pcDNA3.1(+)) /well. Next, 100 μL 3-(4,5-dimethylthiazol-2-yl)-2,5-diphenyltetrazolium bromide (MTT) (EMD Biosciences, San Diego, CA, USA) was added to each well and incubated for 2 h, 4 h, and 1, 2, 3, 4 days after transfection. The absorbance was then measured using an enzyme-linked immunosorbent assay (ELISA) reader (Thermo scientific) at 570 nm. The cell growth rate was normalized to the absorbance measured at 4 h.

### Colony formation assay

MDA-MB-231 cells were seeded in six-well plate at a density of 500 cells/well. After incubation for 2 weeks, cells were fixed with a solution containing 75% methanol and 25% acetate (Sigma) and stained with 0.1% crystal violet (Sigma). Colonies with a cell number greater than 50 were counted and quantified using ImageJ v1.51 software.

### Wound healing assay

MDA-MB-231 cells were seeded in the well of the Ibidi Culture-Insert (Ibidi, Martinsried, Germany) at a density of 4.5 × 10^4^ cells/well overnight. The following day, the culture inserts were removed gently with sterile tweezers to create a cell-free gap. The gap area was imaged on the microscope at 0 and 24 h and quantified using ImageJ software.

### Tube formation assay

HUVECs were starved in serum-free DMEM for 2 h, then resuspended in a fresh conditioned medium, and seeded onto a 96-well plate coated with 50 μl Matrigel (R&D Systems, Bio-Techne, MN, USA) at a density of 2.2 × 10^4^ cells/well. Conditioned media were collected by incubating MDA-MB-231 cells transfected with pcDNA3.1(+)-*NDRG1-OT1* or empty vector (pcDNA3.1(+)) with serum-free DMEM for 24 h, and concentrated by centrifuging at 4400 × *g* for 13 min with an Amicon Ultra-4 Centrifugal Filter Unit (Millipore). The capability of tube formation was viewed via microscope at 2.5 and 6 h, and quantified using ImageJ software.

### Transwell invasion assay

MDA-MB-231 cells were seeded in the upper chamber of Corning Costar Transwell cell culture inserts (Merck, Darmstadt, Germany) coated with 20 μL 25% Matrigel (R&D Systems) at 1 × 10^5^ cells in 100 μL of serum-free medium. The lower chamber was filled with 600 μL DMEM containing 10% FBS. After 48 h of incubation, MDA-MB-231 cells were fixed with a fixing solution containing 75% methanol and 25% acetate (Sigma) for 10 min, and cells on the upper side of the membrane surface were removed by scraping with a cotton swab. The cells that passed through the filter were stained with 0.1% crystal violet (Sigma) for 10 min. The cell migration and invasion ability of the stained cells were captured by microphotograph and measured using ImageJ software.

### Xenografts and bioluminescence imaging

The animal studies, including the number of animals and exclusion criteria, were approved by the Institutional Animal Care and Use Committee (IACUC) of National Taiwan University College of Medicine Laboratory Animal Center (Taipei, Taiwan) (Approval numbers: 20160307). All mice were given water and irradiated rodent chow *ad libitum*. MDA-MB-231-luc cells transfected with pcDNA3.1(+)-*NDRG1-OT1* or empty vector (pcDNA3.1(+)) were subcutaneously injected into the lower back of 7-week-old female SCID mice purchased from the Laboratory Animal Center (Taipei, Taiwan) at 2 × 10^6^ cells in 50 µL PBS containing 50% Matrigel Basement Membrane Matrix (Scientific Laboratory Supplies Limited, UK). All animals were randomly divided into the treatment group or empty control group, and each group contained four mice. Tumor size was monitored using an In Vivo Imaging System (IVIS; PerkinElmer, MA, USA) after intraperitoneal administration of 200 μL of 15 mg/mL VivoGlo™ Luciferin (Promega, WI, USA) to the mice at 1, 2, 3, 4, 5, 6, and 7 weeks after tumor cell injection. Mice were anesthetized by isoflurane inhalation *via* a nasal cone during whole-body imaging. A region of interest was manually selected over the signal intensity. Luminescence values were expressed in photons per second (p·s^−1^). All bioluminescence data were collected and analyzed using the IVIS. After all, in vivo experiments were completed, the mice were sacrificed, and the tumors were harvested. The mass of each tumor was measured by people who were unaware of the treatment each animal received.

### Immunofluorescence

To identify whether *NDRG1-OT1* encodes peptides, HEK293T cells were seeded at 4 × 10^4^ cells/well on 22 mm circle glass coverslips (FisherBrand, Thermo Fisher, Waltham, MA, USA) in 12-well plates and transfected with pcDNA3.1/ ZEO(+)-*NDRG1-OT*1 plasmids using jetPRIME (Polyplus-transfection) according to the manufacturer’s protocol. After 48 h, cells were fixed with 4% paraformaldehyde (Sigma, Saint Louis, MO, USA), permeabilized with TBST, and blocked with 1% bovine serum albumin (Sigma) for 1 h on a 75 rpm shaker at room temperature. Cells were stained with primary antibodies, anti-FLAG (GeneScript, New Jersey, USA) and anti-HA (Cell Signaling), and secondary IgG antibodies, Alexa Fluor 488 and Cy3-conjugated (Jackson ImmunoResearch, West Grove, PA, USA), for 1 h on a 75 rpm shaker at room temperature. After washing three times with TBST, cellular nuclei were stained with 4’,6-diamidino-2-phenylindole (DAPI) (Sigma). Cells were imaged on a microscope (Zeiss Axio Imager, M1, Zeiss, Oberkochen, Germany) and analyzed by ZEN blue software (Carl Zeiss Microscopy, LLC, White Plains, NY, USA).

### Statistical analysis

All results are presented as means ± SDs for at least three independent experiments to ensure adequate power to detect a prespecified effect and are indicated in the respective figure legends. Data with normal distributions were analyzed by the homogeneity of variance tests and Student’s two-tailed unpaired *t*-test to assess the differences between each group (GraphPad Prism version 6.0, GraphPad Prism Software Inc., CA, USA). *P* values lower than 0.05 were considered to be statistically significant. One asterisk and two asterisks represent *P* < 0.05 and *P* < 0.01, respectively.

## Results

**Fig. 1:**
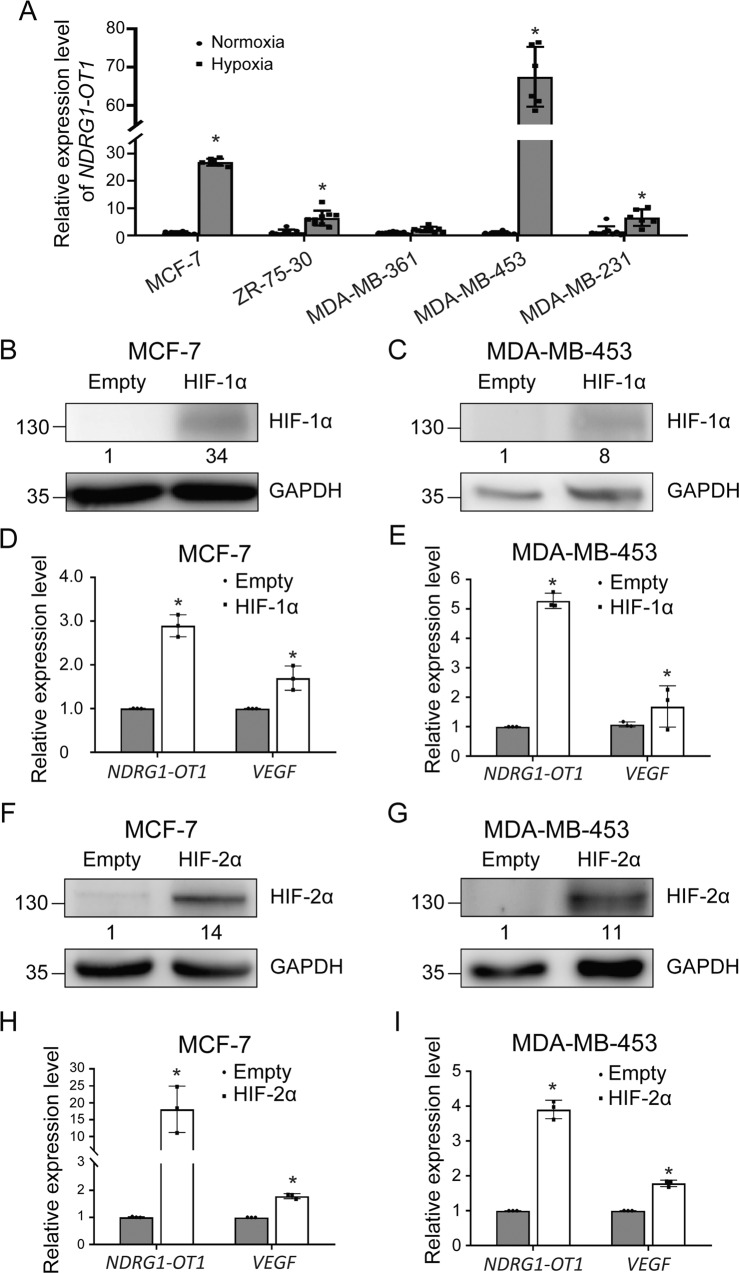
*NDRG1-OT1* is upregulated by HIF proteins under hypoxia in different breast cancer cell lines. **A** Expression levels of *NDRG1-OT1* in five breast cancer cell lines under hypoxia and normoxia, detected by qPCR. Loading control: 18 S rRNA. The relative expression levels in each cell line were compared with those of the normoxic group, respectively. **B**, **C** Western blots of HIF-1α in MCF-7 (**B**) and MDA-MB-453 (**C**) cells overexpressing HIF-1α-P402A/P564A under normoxia. Cells were transfected with 2 μg pcDNA3-HIF-1α-P402A/P564A or empty plasmid. **D**, **E** Expression of *NDRG1-OT1* and *VEGF* in MCF-7 (**D**) and MDA-MB-453 (**E**) cells overexpressing HIF-1α-P402A/P564A under normoxia. **F**, **G** Western blot analysis of HIF-2α in MCF-7 (**F**) and MDA-MB-453 (**G**) cells overexpressing HIF-2α-P405A/P531A under normoxia. Cells were transfected with 2 μg pcDNA3-HIF-2α-P405A/P531A or empty plasmid. **H**, **I** Expression of *NDRG1-OT1* and *VEGF* in MCF-7 (**H**) and MDA-MB-453 (**I**) cells overexpressing HIF-2α-P405A/P531A under normoxia. The results are means ± SDs (*n* = 4). *P < 0.05.

Previously, both *NDRG1* and *NDRG1-OT1* were found to be upregulated in MCF-7 breast cancer cells under hypoxia [29]. We first examined whether *NDRG1-OT1* was affected by overexpressing its host gene, *NDRG1*. As shown in Fig. S1, the expression levels of *NDRG1-OT1* were not affected in cells overexpressing *NDRG1*. To further investigate if the effects of hypoxia on *NDRG1-OT1* were general, we examined the relative expression levels of *NDRG1-OT1* in different breast cancer cell lines classified by their intrinsic molecular subtypes, including MCF-7 (luminal A), ZR-75-30 (luminal B), MDA-MB-361 (luminal B), MDA-MB-453 (HER2^+^), and MDA-MB-231 (triple negative) under hypoxia and normoxia (Fig. 1A). The expression levels of *NDRG1-OT1* were upregulated under hypoxia in all cell lines. These data imply that the hypoxia-driven effects on *NDRG1-OT1* are common in human breast cancer cell lines, and *NDRG1-OT1* might play a role in cellular adaptation to hypoxia.

Since *NDRG1-OT1* was dramatically upregulated under hypoxia, we hypothesized that this effect was regulated by HIFs. In order to mimic hypoxia, we overexpressed a *HIF1A* mutant (pcDNA3-HIF-1α-P402A/P564A), which resists O_2_-regulated prolyl hydroxylation in the oxygen-dependent degradation domain, in MCF-7 (Fig. 1B) and MDA-MB-453 (Fig. 1C) cells under normoxia. When cells were overexpressing the mutant HIF-1α, RNA levels of *NDRG1-OT1* and *VEGF*, HIF-1α regulated gene, were significantly (*P* < 0.05) upregulated in both cell lines (Fig. 1D&E), similar to their response to hypoxia. In addition, since HIF-2α has been implicated in response to hypoxia [31, 32], a *HIF2A* mutant, pcDNA3-HIF-2α-P405A/P531A, was overexpressed in MCF-7 (Fig. 1F) and MDA-MB-453 (Fig. 1G) cells under normoxia, and *NDRG1-OT1* and *VEGF* were again significantly (*P* < 0.05) upregulated in both cell lines (Fig. 1H, I). Taken together, these data suggest that *NDRG1-OT1* can be transcriptionally activated by overexpressing HIF-1α and HIF-2α.

**Fig. 2:**
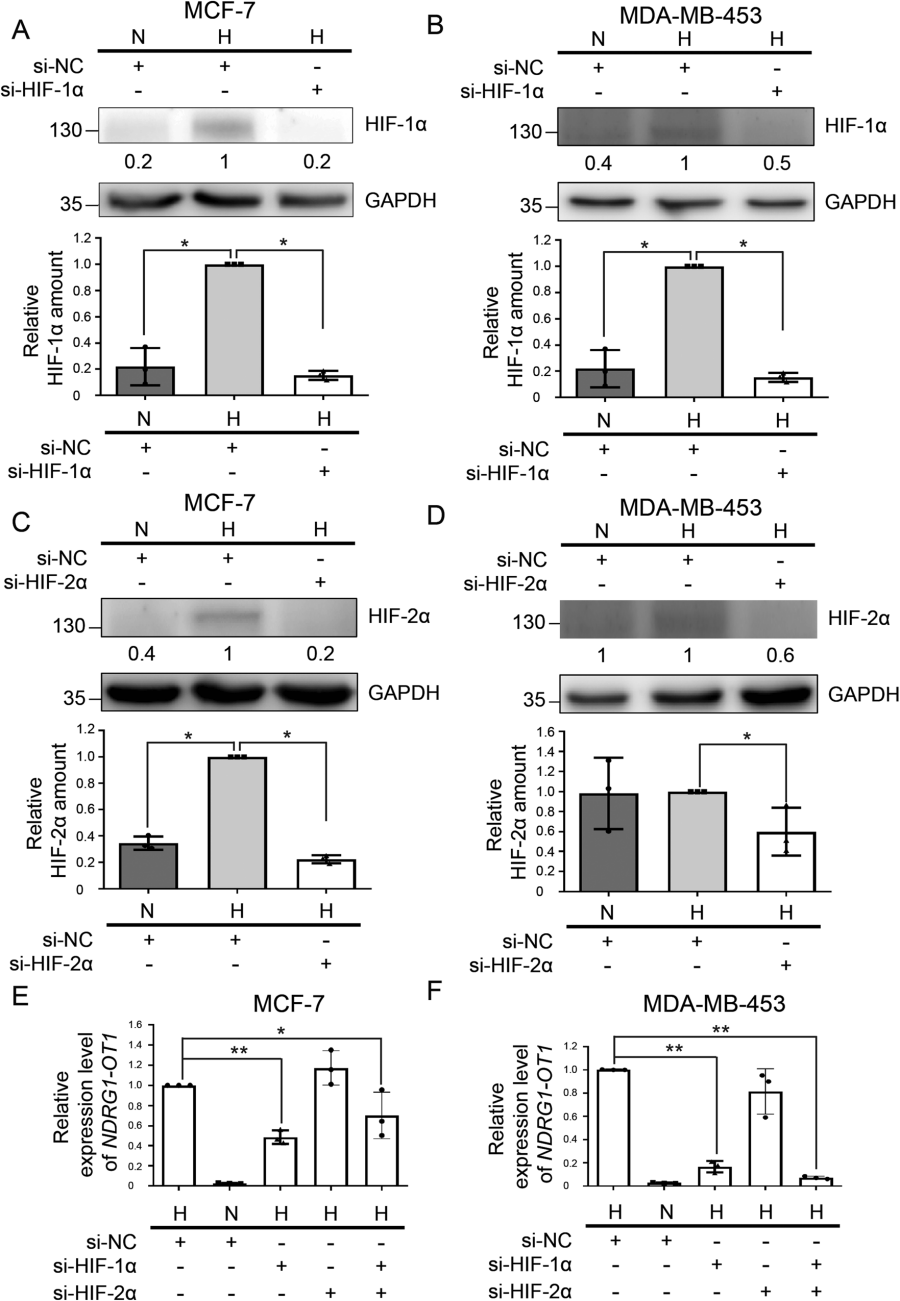
Expression of *NDRG1-OT1* is induced by HIF-1α, not HIF-2α, under hypoxia. **A**, **B** Western blots of HIF-1α in MCF-7 (**A**) and MDA-MB-453 (**B**) cells transfected with 12.5 μM siRNA against *HIF1A* under hypoxia. Top: representative western blots. Bottom: statistical bar chart. Loading control: GAPDH. **C**, **D** Western blots of HIF-2α in MCF-7 (**C**) and MDA-MB-453 (**D**) cells transfected with 12.5 μM siRNA against *HIF2A* under hypoxia. Top: representative western blots. Bottom: statistical bar chart. Loading control: GAPDH. **E**, **F** Relative expression levels of *NDRG1-OT1* in MCF-7 (**E**) and MDA-MB-453 (**F**) cells transfected with siRNA against *HIF1A*, *HIF2A*, or *HIF1A* + *HIF2A* under hypoxia. The relative expression level of each condition was compared with hypoxic group of the negative control (NC), respectively. All data shown are the means ± SDs (*n* = 3). N normoxia. H hypoxia. *P < 0.05. **P < 0.01.

Next, to explore the regulatory mechanism of *NDRG1-OT1* under hypoxia, we further knocked down HIFs under hypoxia to explore which HIF could diminish *NDRG1-OT1* expression. The protein levels of HIF-1α (Fig. 2A, B) and HIF-2α (Fig. 2C, D) were ablated under hypoxia by transfecting cells with 4 siRNAs against each protein. Surprisingly, knockdown of *HIF1A* or both *HIF1A* and *HIF2A*, but not *HIF2A* alone, significantly (*P* < 0.05) attenuated *NDRG1-OT1* expression under hypoxia (Fig. 2E, F). These results suggest that only HIF-1α activates the transcription of *NDRG1-OT1*.

**Fig. 3:**
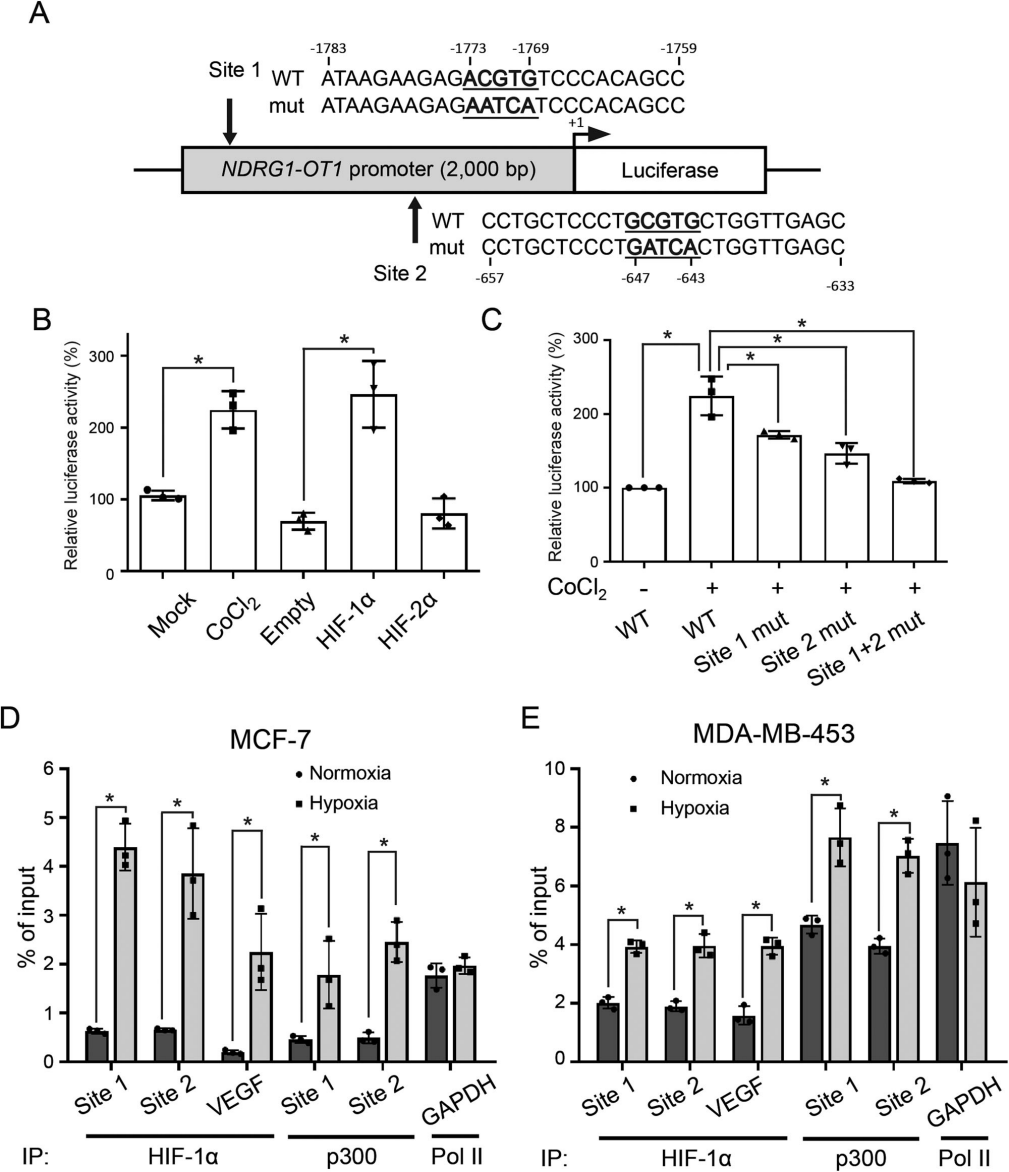
HIF-1α and p300 physically binds to the promoter of *NDRG1-OT1*. **A** Schematic representation of firefly luciferase constructs containing the *NDRG1-OT1* promoter (−2000 to −1 bp), showing both the wild-type sequence (WT) and the mutation of the HIF-1α binding sites (mut). There are two putative HREs ([A/G]CGTG) within the promoter of *NDRG1-OT1*. Site 1: −1773 to −1769 bp; site 2: −647 to −643 bp. **B** Luciferase reporter assays of *NDRG1-OT1* promoter in cells treated with CoCl_2_, or overexpressing HIF-1α or HIF-2α. HEK293T cells were transfected with HIF-1α or HIF-2α expressing plasmids, firefly luciferase plasmids, and Renilla reporter vectors. The CoCl_2_ treatments were administered for 24 h. The relative firefly luciferase activity was measured and normalized to Renilla luciferase activity. **C** Luciferase reporter assays of *NDRG1-OT1* mutant promoters in cells treated with CoCl_2_. HEK293T cells were transfected with indicated firefly plasmids and Renilla reporter vectors. The CoCl_2_ treatments were administered for another 24 h after transfection of plasmids for 24 h. **D**, **E** ChIP of HIF-1α, p300, and *NDRG1-OT1* promoter in MCF-7 (**D**) and MDA-MB-453 (**E**) cells under hypoxia. Anti-HIF-1α or anti-p300 antibody was used to precipitate chromatin, and DNA was examined by primers flanking site 1 or site 2 of the *NDRG1-OT1* promoter. *VEGF* promoter served as a positive control. Chromatin precipitated by anti-RNA polymerase II antibody was amplified by primers flanking the *GAPDH* promoter and served as a negative control. The relative chromatin enrichment (%) was normalized to input. All data shown are the means ± SDs (*n* = 3). IP immunoprecipitation. *P < 0.05.

We next investigated how HIF-1α up-regulates *NDRG1-OT1* expression by using luciferase reporter assays. Promoter analysis revealed that there were two putative HREs ([A/G]CGTG) located at −1773 to −1769 (site 1) and −647 to −643 bp (site 2) relative to the transcription start site of *NDRG1-OT1* (Fig. 3A). Therefore, the promoter region of *NDRG1-OT1* (−2000 to −1 bp) was inserted into the pGL3-basic vector and fused with the luciferase gene. Both overexpression of HIF-1α and treatment with the hypoxia mimetic compound, CoCl_2_ [33], increased luciferase activity (Fig. 3B), and overexpression of HIF-2α could not increase the luciferase activity (Fig. 3B). To identify the role of the two putative HREs, the HRE sequences were mutated (Fig. 3A). The mutation of either site 1 or site 2 decreased the luciferase activity, and mutation of both sites suppressed the promoter activity to a greater extent (Fig. 3C). To further understand the roles of the two HREs on the promoter-driven expression of *NDRG1-OT*1 under hypoxia, ChIP-qPCR assays were performed to validate the direct interaction between HIF-1α and the two HREs. The chromatin precipitated by anti-HIF-1α antibody was amplified by qPCR using primers flanking site 1 or site 2 (Fig. 3D, E). In addition, as hypoxia-induced target activation is mediated by the HIF-1 transcriptional complex, composed of HIF-1 heterodimer and transcriptional cofactor p300/CBP [34], chromatin was also immunoprecipitated by anti-p300 antibody (Fig. 3D, E). Significantly, the ChIP-qPCR assays using anti-HIF-1α and anti-p300 antibodies showed that these transcription factors were enriched on the target promoters in hypoxic cells (Fig. 3D, E). These data indicate that HIF-1α and its transcriptional cofactor p300/CBP might be responsible for upregulating *NDRG1-OT1* under hypoxia by directly binding to the HREs of the *NDRG1-OT1* promoter.

**Fig. 4:**
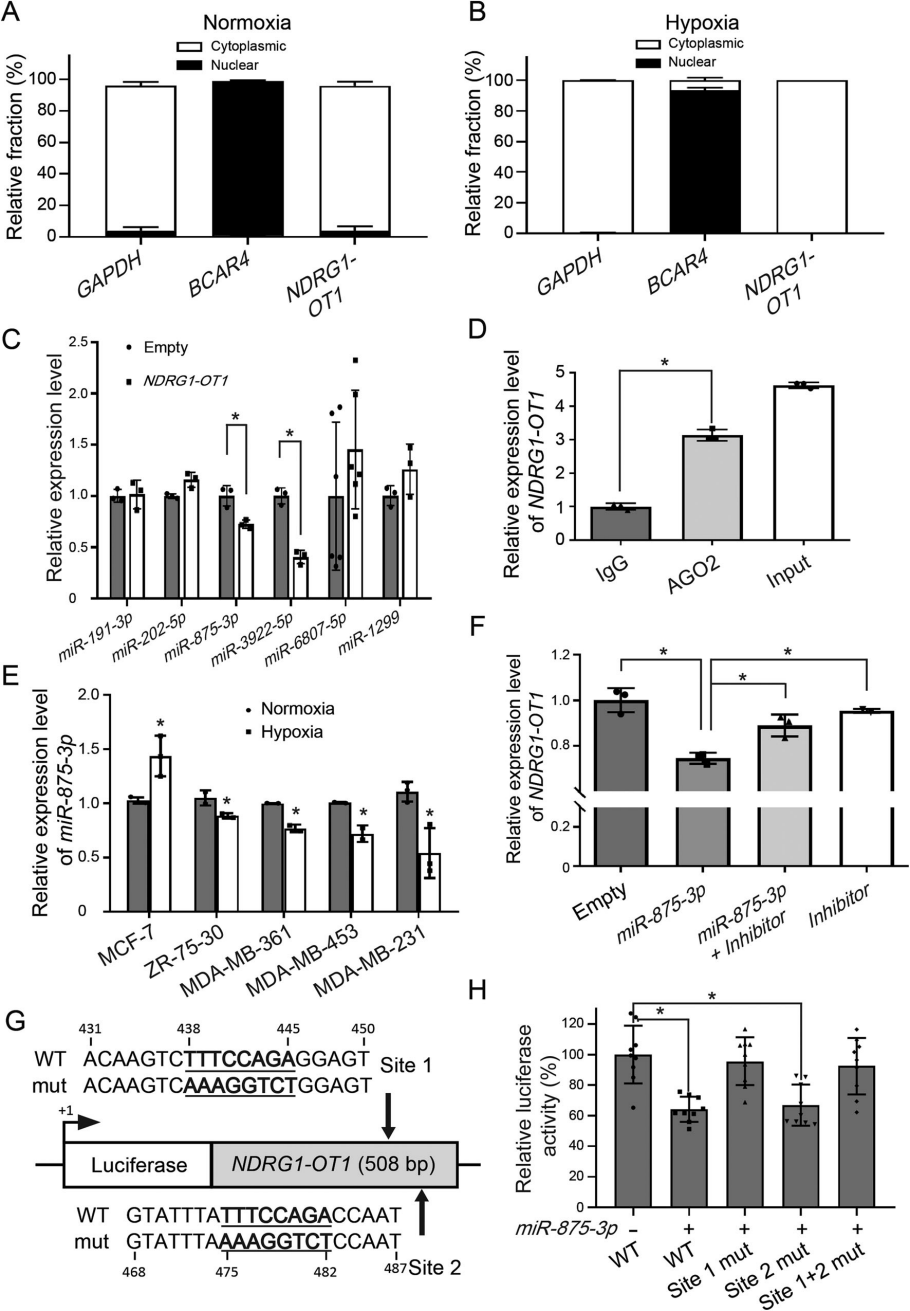
*NDRG1-OT1* serves as a sponge for *miR-875-3p*. **A**, **B** Distribution of *NDRG1-OT1* in MDA-MB-231 cells under normoxia (**A**) and hypoxia (**B**). Cytoplasmic and nuclear RNA were fractionated in MDA-MB-231 cells under normoxia (**A**) and hypoxia (**B**). Relative abundance of RNA was normalized to the total amount of RNA and detected by qPCR. *GAPDH*: cytoplasmic marker; *BCAR4*: nuclear marker. **C** Relative expression levels of six predicted miRNAs in MDA-MB-231 cells overexpressing *NDRG1-OT1*. The candidate miRNAs binding *NDRG1-OT1* were predicted by miRDB (http://mirdb.org/). The expression levels of miRNAs were measured by qPCR and normalized to *U6*. **D** RIP analysis of *NDRG1-OT1* using antibody against AGO2 in normoxia. The RIP enrichment of the AGO2-associated lncRNA was measured by qPCR and normalized to 18 S rRNA. The relative fold enrichment was calculated as compared to the IgG group. **E** Expression levels of *miR-875-3p* in five breast cancer cell lines under hypoxia and normoxia, detected by qPCR. Loading control: *U6* snRNA. The relative expression levels in each cell line were compared with those of the normoxic group, respectively. **F** Relative expression of *NDRG1-OT1* in MDA-MB-231 cells overexpressing *miR-875-3p*, and followed by treatment of *miR-875-3p* inhibitor (50 nM). Loading control: *GAPDH*. (*n* = 5). **G** Schematic representation of firefly luciferase constructs containing the sequence of *NDRG1-OT1* (WT) and two mutations of *miR-875-3p* binding sites (sites 1 and 2). **H** Luciferase reporter assays of *NDRG1-OT1* in HEK293T cells overexpressing *miR-875-3p*. All data shown are the means ± SDs. *P < 0.05.

Next, to determine whether *NDRG1-OT1* could serve as a miRNA sponge to modulate cell functions, we first examined the distribution of *NDRG1-OT1* in cells under normoxia (Fig. 4A), and hypoxia (Fig. 4B). Cell fractionation analysis showed that *NDRG1-OT1* was present mostly in the cytoplasmic fraction (Fig. 4A, B). Next, in silico analysis using miRDB [35] was performed to predict the miRNAs targeting *NDRG1-OT1*. The top ten miRNAs, whose seed regions matched the *NDRG1-OT1* sequence (Table 1), were predicted. To validate whether *NDRG1-OT1* could act as a sponge for these miRNAs, qPCR of six randomly selected miRNAs was performed. The expression levels of these miRNAs were examined in MDA-MB-231 cells overexpressing *NDRG1-OT1*. Only *miR-875-3p* and *miR-3922-5p* were significantly (*P* < 0.05) downregulated (Fig. 4C). Next, since AGO2, an essential component of the RNA-induced silencing complex (RISC), is responsible for messenger RNA cleavage activity exerted by miRNAs [36], RIP assays using anti-AGO2 antibody were performed and followed by qPCR. As shown in Fig. 4D, *NDRG1-OT1* was enriched in AGO2-immunoprecipitated complexes as compared with the IgG control group. *MiR-875-3p* was chosen for performing the following experiments because *miR-875-3p* was predicted with the highest possibility to sponge with *NDRG1-OT1* (Table 1) and had higher endogenous expression levels than *miR-3922-5p*. In addition, the expression levels of *miR-875-3p* were significantly downregulated in hypoxia in most breast cancer cell lines, except MCF-7 cells (Fig. 4E).

**Table 1:** The predicted *NDRG1-OT1* sponged miRNAs by miRDB.

Target rank	Target score	miRNA name	PCR validation^a^
1	85	*miR-875-3p*	O
2	78	*miR-3922-5p*	O
3	74	*miR-202-5p*	X
4	68	*miR-6807-5p*	X
5	60	*miR-7107-3p*	
6	60	*miR-6753-3p*	
7	60	*miR-4527*	
8	59	*miR-6503-5p*	
9	57	*miR-191-3p*	X
10	57	*miR-1299*	X

^a^O: miRNAs could be validated; X: miRNAs could not be validated.

First, to examine whether there was a reciprocal inhibition between *NDRG1-OT1* and *miR-875-3p*, the expression levels of *NDRG1-OT1* were examined in MDA-MB-231 cells overexpressing *miR-875-3p*, and followed by *miR-875-3p* inhibitor. The results showed that *NDRG1-OT1* could be significantly inhibited by *miR-875-3p*, and released from the inhibition by the additional treatment of *miR-875-3p* inhibitor or *miR-875-3p* inhibitor alone (Fig. 4F). To validate this phenomenon, luciferase reporter assays were performed. Since two potential binding sites, 438–445 bp (site 1) and 475–482 bp (site 2), of *miR-875-3p* were identified in *NDRG1-OT1*, mutants of these sites were constructed to examine whether *miR-875-3p* could still bind to *NDRG1-OT1* (Fig. 4G). Similar to the results of the expression assays, the luciferase activity decreased in cells overexpressing *miR-875-3p*. Only mutation in site 1 (438–445 bp) alone or in combination with site 2 (475–482 bp), but not site 2 alone, could reverse the inhibition of luciferase activity (Fig. 4H), indicating that *miR-875-3p* binds to *NDRG1-OT1* at site 1. Taken together, these data indicate that *NDRG1-OT1* can serve as a miRNA sponge to inhibit the effects of *miR-875-3p*, and that *miR-875-3p* can also inhibit the expression of *NDRG1-OT1*.

**Fig. 5:**
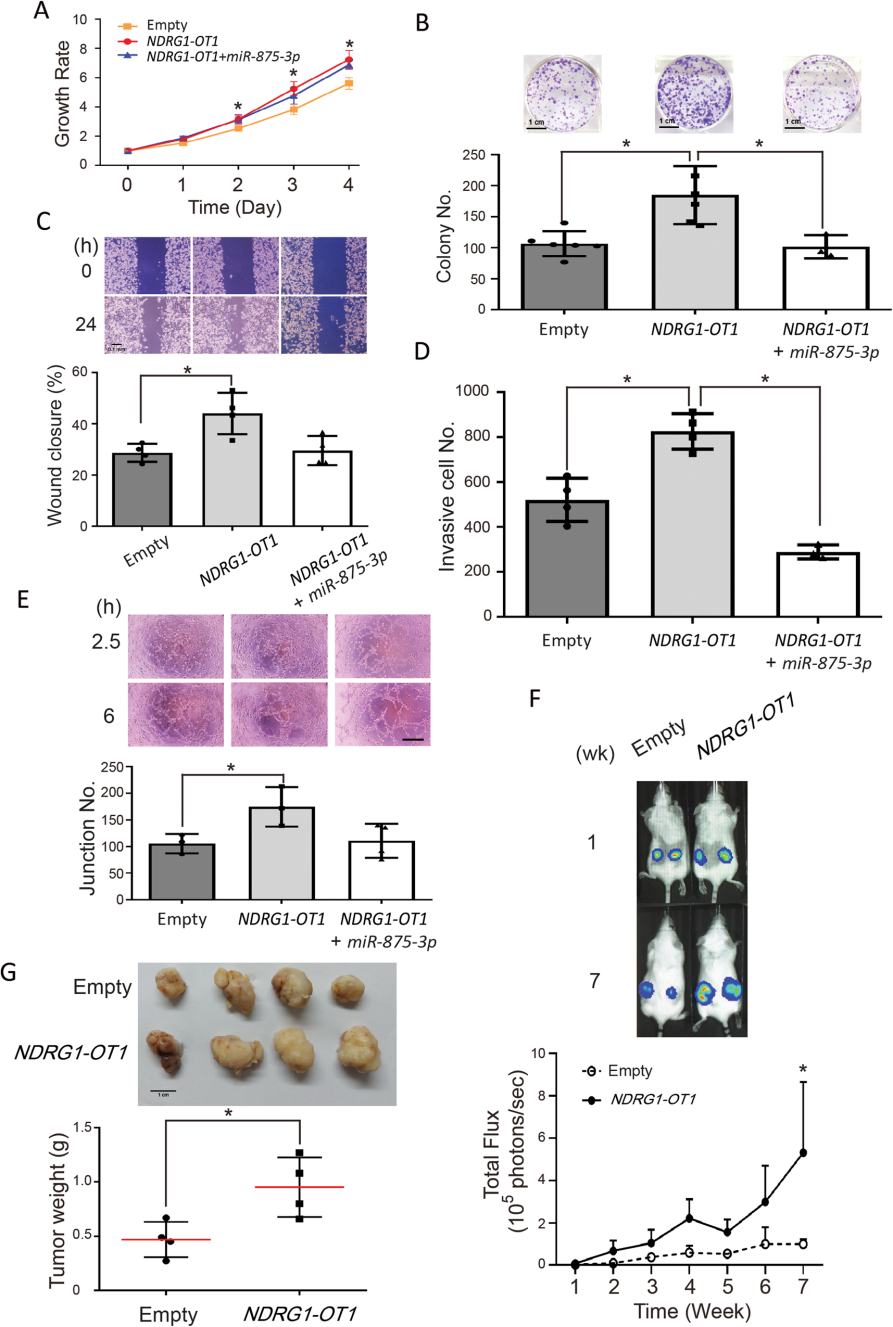
*NDRG1-OT1* promotes tumor malignancy of triple-negative breast cancer MDA-MB-231 cells via inhibiting *miR-875-3p*. **A** MTT assays of MDA-MB-231 cells overexpressing *NDRG1-OT1* or *NDRG1-OT1* + *miR-875-3p* versus empty vector. Proliferation assays were performed by adding MTT at different time points. **B** Colony formation assays. Top: representative picture; bottom: quantification of colony number. **C** Wound healing assays. Top: representative pictures taken at 0 and 24 h; bottom: quantification of wound closure at 24 h relative to 0 h. Scale bar: 0.1 mm. **D** Transwell invasion assays. Cells were seeded after 24 h of transfection. Invasion ability was measured at 48 h after seeding. **E** Tube formation assay. Angiogenesis was measured in HUVECs incubated with conditional medium from *NDRG1-OT1*-overexpressing MDA-MB-231 cells or co-transfecting *miR-875-3p*. Top: representative pictures taken at 2.5 and 6 h; bottom: quantification of junction number at 24 h. Scale bar: 1 mm. **F** Xenograft assays of MDA-MB-231 cells overexpressing *NDRG1-OT1* in SCID mice for 7 weeks using the IVIS imaging system. Top: Photograph of bioluminescence imaging after implantation; bottom: quantification of total flux (photons/sec) (*n* = 4). **G** Photograph and weight of tumors after dissection. Top: Photograph of tumor size at 7 weeks after implantation; bottom: quantification of tumor weight (*n* = 4). *P < 0.01.

Since tumors become more malignant in hypoxia [37, 38], we examined the effects of *NDRG1-OT1* in a more common triple-negative breast cancer cell line, MDA-MB-231, which has a shorter doubling time (doubling time: 25 h) than MDA-MB-453 cells (doubling time: 38 h), based on empirical reasons. Overexpression of *NDRG1-OT1* caused significantly increased growth rates (Fig. 5A) and enhanced colony formation (Fig. 5B) in MDA-MB-231 cells. In addition, overexpression of *NDRG1-OT1* significantly enhanced the migration (Fig. 5C) and invasion (Fig. 5D) ability of MDA-MB-231 cells. Next, we studied whether *NDRG1-OT1* could induce angiogenesis in peripheral endothelial cells. Conditioned culture medium from *NDRG1-OT1*-overexpressing MDA-MB-231 cells was collected and used to incubate HUVECs. After a 6 h incubation, the number of junctions was significantly increased in HUVECs cultured with *NDRG1-OT1* conditioned medium (Fig. 5E), suggesting *NDRG1-OT1* promoted the release of angiogenic factors from triple-negative breast cancer cells.

Furthermore, once overexpression of both *NDRG1-OT1* and *miR-875-3p* in MDA-MB-231 cells, the enhanced malignancy was inhibited by showing a significant reduction in colony formation (Fig. 5B), migration (Fig. 5C), and invasion (Fig. 5D) ability of MDA-MB-231 cells. Also, the number of junctions was not significantly increased in HUVECs cultured with *NDRG1-OT1* + *miR-875-3p* conditioned medium (Fig. 5E).

**Fig. 6:**
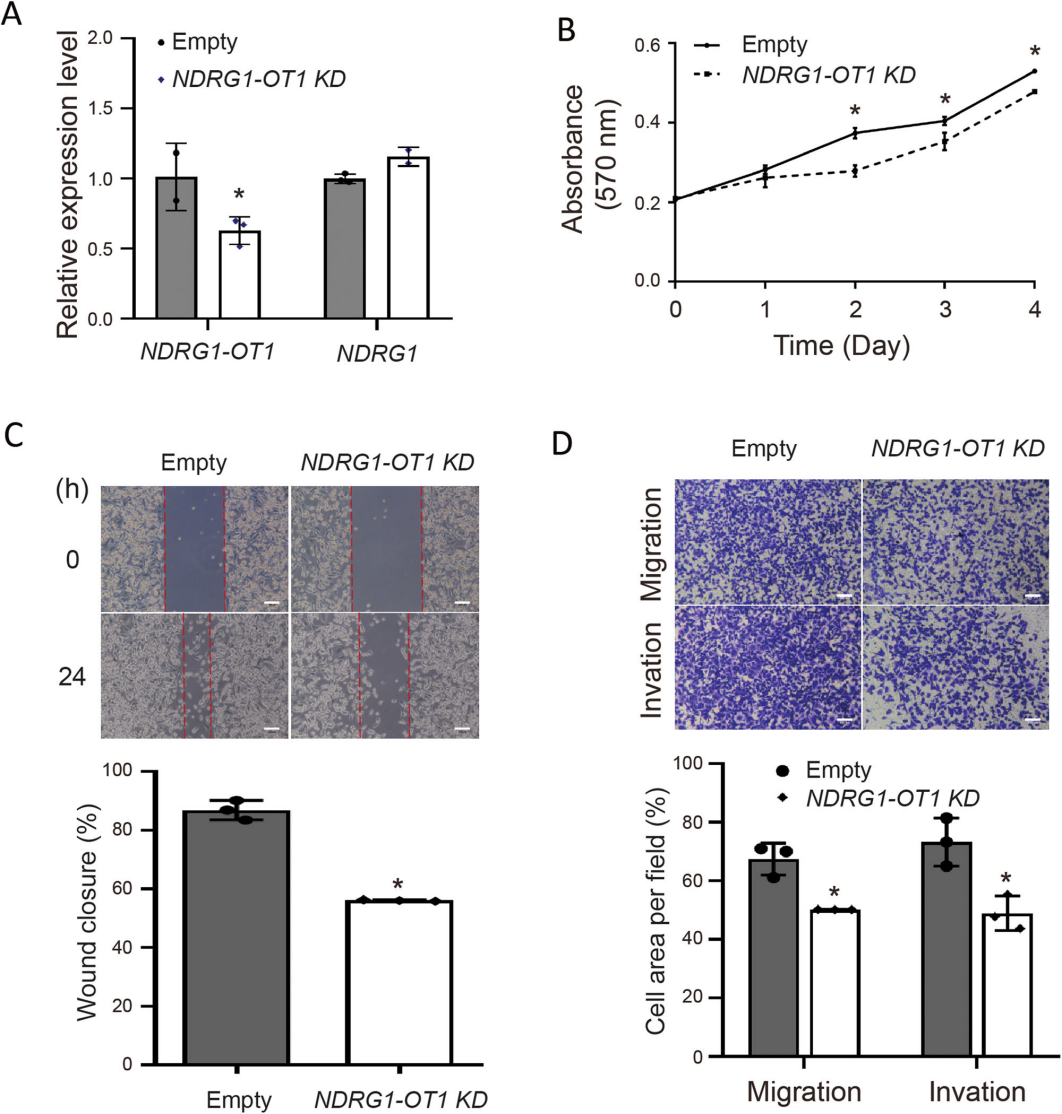
Knockdown of *NDRG1-OT1* inhibits tumor malignancy of triple-negative breast cancer MDA-MB-231 cells. **A** Relative expression levels of *NDRG1-OT1* and *NDRG1* in MDA-MB-231 cells after knocked down *NDRG1-OT1* by Gapmer antisense oligonucleotides. **B** MTT assays of MDA-MB-231 cells after knockdown *NDRG1-OT1*. Proliferation assays were performed by adding MTT at different time points. **C** Wound healing assays. Top: representative pictures taken at 0 and 24 h; bottom: quantification of wound closure at 24 h relative to 0 h. Scale bar: 0.1 mm. **D** Transwell migration and invasion assays. Migration ability was measured at 24 h after seeding. Invasion ability was measured at 48 h after seeding. Scale bar: 100 μm. *P < 0.05.

Next, stable clones of MDA-MB-231-luciferease cells with overexpression of *NDRG1-OT1* were subcutaneously injected into the lower back of 7-week-old female SCID mice. After injection, the signals of luciferase were measured and quantified by IVIS every week. The tumors overexpressing *NDRG1-OT1* were significantly bigger (Fig. 5F) and heavier (Fig. 5G) than negative control tumors. Conversely, when *NDRG1-OT1* were specifically knocked down by antisense oligonucleotide (Fig. 6A), the ability of proliferation (Fig. 6B), migration (Fig. 6C), and invasion (Fig. 6D) in triple-negative breast cancer MDA-MB-231 cells were significantly (*P* < 0.05) decreased. These data revealed that *NDRG1-OT1* promotes cell proliferation, colony formation, migration, and invasion in triple-negative breast cancer cells, and angiogenesis in peripheral endothelial cells.

**Fig. 7:**
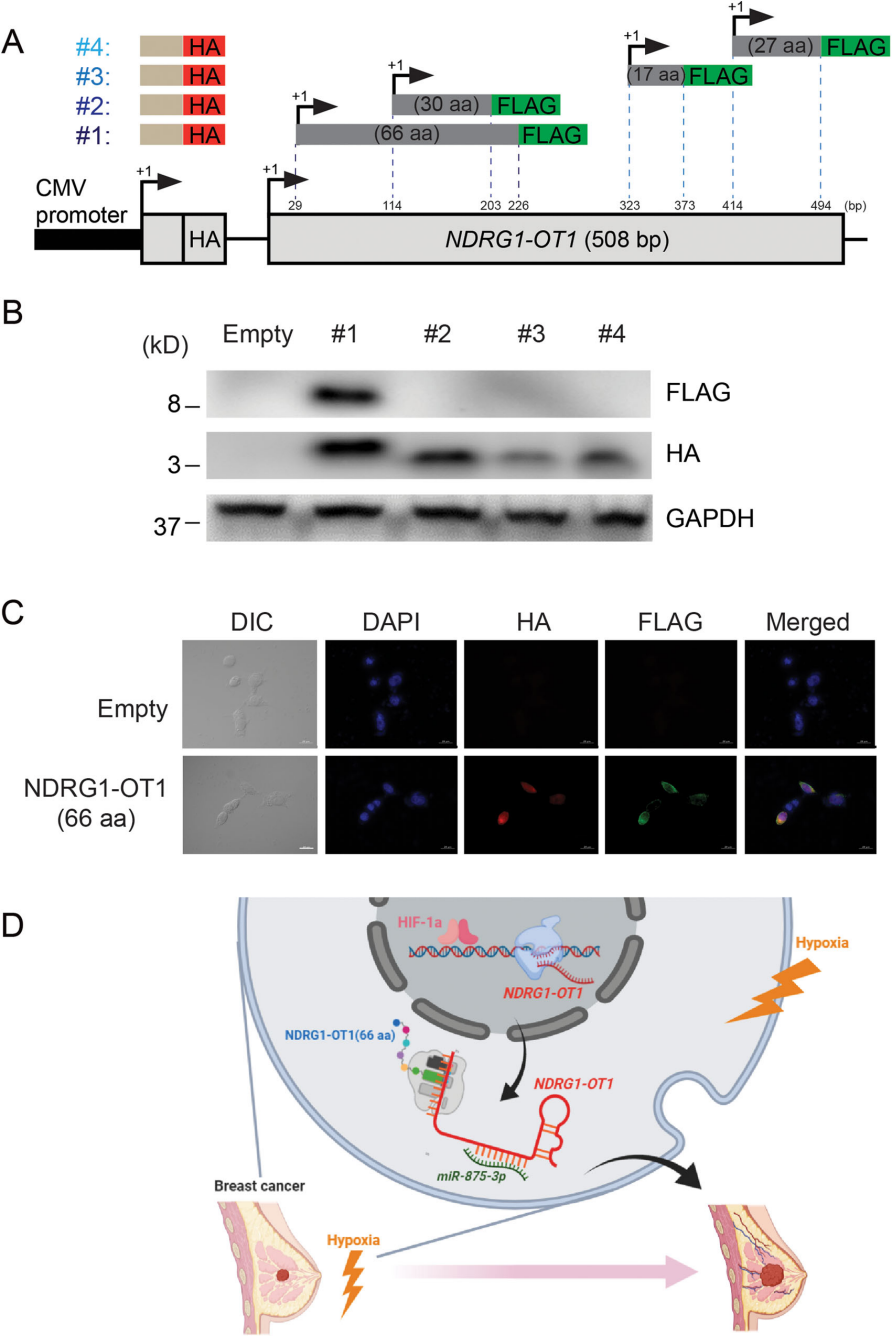
*NDRG1-OT1* encodes a small peptide of 66 amino acids. **A** Schematic representation of FLAG-tagged constructs of four predicted translatable regions. The HA tag was incorporated into the CMV promoter of each plasmid. **B** Western blots of HEK293T cells transfected with FLAG-tagged *NDRG1-OT1* plasmids from panel **A**. FLAG: NDRG1-OT1; HA: translation positive control; GAPDH: loading control. **C** Immunofluorescence staining of NDRG1-OT1 (66 a.a.). DIC: differential interference contrast microscopy; DAPI: nucleus marker. Scale bar: 20 μm. **D** A proposed model for illustrating the regulatory mechanisms and function of hypoxia-induced lncRNA *NDRG1-OT1* in breast cancer cells.

Although lncRNA was originally considered not to encode a protein, there have been more and more reports of the translational capability of lncRNA in recent years [39–41]. To investigate whether *NDRG1-OT1* could be translated into peptides, we transfected 4 FLAG-tagged constructs of predicted translatable regions into HEK293T cells (Fig. 7A). A start codon and the HA tag were inserted in front of each predicted translatable region as a positive control for translation (Fig. 7A). As shown in Fig. 7B, the results of western blotting indicated that all plasmids were transfected successfully but only the #1 construct (29–226 bp, 66 aa) was translated into a peptide (Fig. 7B). Immunofluorescence staining data also revealed that the fluorescent signaling of the FLAG tag was detected and colocalized with the HA tag in HEK293T cells transfected with pcDNA3.1/ZEO(+)-*NDRG1-OT*1 #1 plasmids (Fig. 7C), indicating that the region of *NDRG1-OT1* between 29 and 226 bp could be translated into a small peptide. Collectively, these results illustrated that hypoxia-responsive long noncoding *NDRG1-OT1* could be transcriptionally activated by HIF-1α, act as a miRNA sponge of *miR-875-3p*, translate a small peptide (66 a.a.), and promote tumor growth and migration in breast cancer cells (Fig. 7D).

## Discussion

In this study, we demonstrated that *NDRG1-OT1* was upregulated under hypoxia in different breast cancer cell lines by HIF-1α, but not HIF-2α. Next, luciferase reporter assays and ChIP-qPCR assays showed that HIF-1α transcriptionally activated *NDRG1-OT1* through directly binding to HREs in its promoter region. Thirdly, expression profiling of predicted miRNAs and RIP assays against AGO2 suggested that *NDRG1-OT1* could serve as a miRNA sponge for *miR-875-3p*. Functional assays showed that *NDRG1-OT1* could enhance tumor malignancy. Lastly, the lncRNA *NDRG1-OT1* may encode a small peptide of 66 amino acids.

Over the past few years, a number of studies have shown that the expression of a specific group of lncRNAs is modulated in tumors under hypoxia and contributes to the proliferation of malignant cells. We found that *NDRG1-OT1* was upregulated under hypoxia in different breast cancer cell lines (Fig. 1), which is consistent with other studies [42–44]. For example, *NEAT1*, a hypoxia-induced nuclear lncRNA, played a pro-tumorigenic role in breast cancer by accelerating cell proliferation and reducing apoptosis [42]. Another hypoxia-responsive lncRNA, *lincRNA-p21*, modulated the Warburg effect by disrupting the VHL-HIF-1α interaction [43]. The lncRNA *linc-RoR*, whose expression was increased in hypoxic regions within tumor cell xenografts in vivo, regulated the hypoxic response through a miR-145/HIF-1α signaling module [44]. These hypoxia-regulated lncRNAs participated in various tumorigenic processes to maintain cellular homeostasis, thus enabling adaptive survival under hypoxic stress environments.

The cellular response to hypoxia is mainly administered by HIF family transcription factors. While HIF-1 plays a major role in controlling the ubiquitous response toward hypoxia, it is clear that a quantity of other transcription factors is also involved, directly or indirectly. For example, hypoxia has been demonstrated to activate NFκB in previous studies [45, 46], and the transcription factors CREB, AP-1, and p53 have also been shown to be regulated by hypoxia [47–49]. However, the expression of most hypoxia-responsive lncRNAs is regulated through HIF, either directly or indirectly [50]. Given the relevance of HIF pathways in tumor progression and the pivotal roles of lncRNA in gene regulation, we explored whether HIF transcription factors were involved in the transcriptional mechanism of *NDRG1-OT1*. HIF-2α, unlike ubiquitously expressed HIF-1α, is mainly expressed in endothelial cells [51]. In this study, the *NDRG1-OT1* promoter region encompassing −2000 to −1 bp relative to the transcription start site (chr8:133,244,504-133,246,503) was extracted for Luciferase reporter assays. Since this region is still in the coding region of *NDRG1*, not the promoter region [52], the HIF family transcription factors regulating *NDRG1* and *NDRG1-OT1* should be different. Furthermore, we explained that ectopic expression of HIF-1α and HIF-2α increasing *NDRG1-OT1* expression (Fig. 1B–E) was because both of them share the same binding motifs (the HREs; [A/G]CGTG). Yet, the knockdown assays of HIFs showed that only knockdown of *HIF1A* or *HIF1A* + *HIF2A*, but not *HIF2A*, significantly (*P* < 0.01) downregulated *NDRG1-OT1* expression under hypoxia (Fig. 2E&F). The expression levels of *NDRG1-OT1* were not affected in hypoxic cells deprived of HIF-2α because functional HIF-1α was still present, indicating that the hypoxia-induced effect on *NDRG1-OT1* was through HIF-1α rather than HIF-2α (Fig. 2E, F). This HIF-1α-mediated effect on *NDRG1-OT1* was similar to most hypoxia-induced lncRNAs described previously [50].

It has recently become apparent that there are mutual regulatory influences between miRNAs and lncRNAs. Firstly, miRNAs can modulate the abundance of lncRNAs via miRNA-triggered lncRNA decay. For instance, the recruitment of let-7 by HuR caused decreased stability of the lncRNA *HOTAIR* [53]. Secondly, lncRNAs can act as miRNA sponges/decoys to inhibit the levels and function of miRNAs. *PTENP1*, an example of a miRNA sponge, regulated tumor suppressor PTEN by acting as a decoy for *PTEN* mRNA-targeting miRNAs [54]. Thirdly, lncRNAs can also compete with miRNAs for binding to target mRNAs. For example, *BACE1AS*, a lncRNA that bears a *miR-485-5p* site, promoted stabilization of *BACE1* by preventing access of *miR-485-5p* to the *BACE1* transcript, which would normally promote its degradation. Lastly, some lncRNAs have the potential to generate miRNAs. For instance, lncRNA *linc-MD1* generates *miR-206* and *miR-133b* from an intron and an exon, respectively [55]. In summary, an expanding body of evidence reveals that lncRNAs and miRNAs work cooperatively to mediate gene expression via complex post-transcriptional mechanisms. Therefore, we investigated the potential for *NDRG1-OT1* to interact with miRNA in cells.

Although different fragments of *NDRG1-OT1* via recruiting distinct proteins had different effects on *NDRG1* transcription in MCF-7 breast cancer cells under hypoxia [30], we discovered that *NDRG1-OT1* was almost always located in a cytoplasmic fraction in MDA-MB-231 cells under both normoxia and hypoxia, which provides ample opportunity for lncRNA-miRNA interaction (Fig. 4A, B). The discrepancy may be that *NDRG1-OT1* executes divergent regulation in different breast cancer cells. In silico analyses predicted the interacting miRNAs (Table 1), and the expression of *miR-875-3p* was decreased in cells overexpressing *NDRG1-OT1* (Fig. 4C). *miR-875-3p* has been reported as a tumor suppressor in prostate cancer [56], colorectal cancer [57], and hepatocellular carcinoma [58]. Down-regulation of *miR-875-3p* in this study suggested a similar role in breast cancer. Conversely, overexpression of *miR-875-3p* also inhibited the expression of *NDRG1-OT1* via the binding site at 438–445 bp (Fig. 4F–H), indicating a reciprocal negative regulation between *NDRG1-OT1* and *miR-875-3p*. Taken together, these results showed mutual inhibition effects between *NDRG1-OT1* and *miR-875-3p*.

Lastly, although lncRNA is traditionally defined as RNA that cannot translate to peptides, recent studies based on ribosome profiling have provided important clues to the unanticipated translation potential of lncRNAs [59, 60]. Ribosome profiling, also known as the high-throughput sequencing of ribosome-protected fragments (Ribo-seq), examined the ribosome association of candidate transcripts and identified many translated genomic regions [61]. Recently, many studies have repeatedly reported that some lncRNAs showed a strong association with ribosomes [41, 62, 63], and a large number of high-quality peptides translated from lncRNAs were identified by mass spectrometry [60]. Although the majority of peptides encoded by lncRNAs are not conserved, the lack of conservation does not preclude the biological importance of lncRNA-encoded peptides [60]. Recently, a few studies confirmed that some lncRNAs could code for a short peptide with key biological functions [64, 65]. For example, a peptide encoded by lncRNA *HOXB-AS3* suppressed the growth of colon cancer [41]. A peptide encoded by lncRNA *DWORF* enhanced sarco/endoplasmic reticulum Ca^2+^-ATPase (SERCA) activity in muscle [63]. A micropeptide, myoregulin, encoded by a putative lncRNA inhibited the pump activity of SERCA from decreasing muscle performance [64]. In this study, we also identified that the coding region (29–226 bp) within *NDRG1-OT1* could be translated into a small peptide of 66 amino acids (Fig. 7B, C). However, when we tried to identify the novel *NDGR1-OT1* peptide under hypoxia. No novel *NDGR1-OT1* peptide was identified by mass spectrometry (data not shown). These results suggested that the scarce amount of this novel *NDGR1-OT1* peptide cannot be detected under natural conditions by mass spectrometry. A more sensitive detecting method is warranted in the future to detect this novel *NDGR1-OT1* peptide.

## Supplementary information

Reproducibility checklist

Supplementary materials

## Data Availability

All data in this paper are available when requested.

## Materials availability

All materials in this paper are available when requested.
