# Supplementary material for: Regulatory mechanisms and function of hypoxia-induced long noncoding RNA NDRG1-OT1 in breast cancer cells
Source: Cell Death Dis. 2022 Sep 20;13(9):807. doi: 10.1038/s41419-022-05253-2 (PMC9489765; doi:10.1038/s41419-022-05253-2)

| **Table S1.** **Primers used for site-directed mutagenesis** | | |
| --- | --- | --- |
| Mutant site | Primer | Sequence* (5’ to 3’) |
| *NDRG1-OT1* -promoter site 1 | Forward | GGCTCTCTGCTTGTCACAATAAGAAGAGAATCATCCCACAGCCGGGGA |
|  | Reverse | TCCCCGGCTGTGGGATGATTCTCTTCTTATTGTGACAAGCAGAGAGCC |
| *NDRG1-OT1* -promoter site 2 | Forward | GGGCATCCTGCTCCCTGATCACTGGTTGAGCTGAGGGC |
|  | Reverse | GCCCTCAGCTCAACCAGTGATCAGGGAGCAGGATGCCC |

* The mutation sites are underlined in the middle of the primers

| **Table S2.** **siGENOME-SMARTpool siRNA sequences** | |
| --- | --- |
| siRNA | Number and sequence (5’ to 3’) |
| siHIF1A SMARTpool | (1) GGACACAGAUUUAGACUUG |
|  | (2) GAUGGAAGCACUAGACAAA |
|  | (3) CGUGUUAUCUGUCGCUUUG |
|  | (4) GAUGAAAGAAUUACCGAAU |
|  |  |
| siHIF2A SMARTpool | (1) GCAAAUGUACCCAAUGAUA |
|  | (2) GAGCGGGACUUCUUCAUGA |
|  | (3) AGACGGAGGUGUUCUAUGA |
|  | (4) ACACAUCUUUGGAUAACGA |
|  |  |
| Non-Targeting siRNA | UAGCGACUAAACACAUCAA |

| **Table S3.** **The primers for reverse transcription and quantitative RT-PCR** | | |
| --- | --- | --- |
| Gene/miRNA | Primer | Sequence (5’ to 3’) |
| **Reverse Transcription** | | |
| *miR-875-3p* | GTTGGCTCTGGTGCAGGGTCCGAGGTATTCGCACCAGAGCCAACCACAACCT | |
| *miR-3922-5p* | GTTGGCTCTGGTGCAGGGTCCGAGGTATTCGCACCAGAGCCAACCACAACCT | |
| *miR-202-5p* | GTTGGCTCTGGTGCAGGGTCCGAGGTATTCGCACCAGAGCCAACCACAACCT | |
| *miR-191-3p* | GTTGGCTCTGGTGCAGGGTCCGAGGTATTCGCACCAGAGCCAACCACAACCT | |
| *miR-6807-5p* | GTTGGCTCTGGTGCAGGGTCCGAGGTATTCGCACCAGAGCCAACCACAACCT | |
| *miR-1299* | GTTGGCTCTGGTGCAGGGTCCGAGGTATTCGCACCAGAGCCAACCACAACCT | |
| *U6* snRNA | CGCTTCACGAATTTGCGTGTCAT | |
| **Quantitative RT-PCR** | | |
| *NDRG1-OT1* | Forward | CTCCCAGGTTCCTGTACTACTG |
|  | Reverse | GGCGGCAGGTAACGAGTCATTG |
| *BCAR4* | Forward | GTTCCGATGCTTGTCTTGCTC |
|  | Reverse | CCAAAGACGAAGATGCCAGG |
| *GAPDH* | Forward | AACGGGAAGCTTGTCATCAATGGAAA |
|  | Reverse | GCATCAGCAGAGGGGGCAGAG |
| *U6* snRNA | Forward | GCTTCGGCAGCACATATACTAAAAT |
|  | Reverse | CGCTTCACGAATTTGCGTGTCAT |
| *miR-875-3p* | Forward | CGCGCCTGGAAACACTG |
|  | Reverse | GTGCAGGGTCCGAGGT |
| *miR-3922-5p* | Forward | CGCGTCAAGGCCAGAGGTC |
|  | Reverse | GTGCAGGGTCCGAGGT |
| *miR-202-5p* | Forward | CCGGCGCGTTCCTATGCATATA |
|  | Reverse | GTGCAGGGTCCGAGGT |
| *miR-191-3p* | Forward | CGGCTGCGCTTGGATTT |
|  | Reverse | GTGCAGGGTCCGAGGT |
| *miR-6807-5p* | Forward | CCCGGTGAGCCAGTGGAA |
|  | Reverse | GTGCAGGGTCCGAGGT |
| *miR-1299* | Forward | CCCGCGTTCTGGAATTCTGTG |
|  | Reverse | GTGCAGGGTCCGAGGT |

| **Table S4.** **Primers used for chromatin immunoprecipitation** | | |
| --- | --- | --- |
| Promoter of gene | Primer | Sequence (5’ to 3’) |
| *NDRG1-OT1*  -promoter site 1 | Forward | GCTCTCTGCTTGTCACAATAAGA |
|  | Reverse | CCATGTGCTCAGATGAACTTG |
| *NDRG1-OT1*  -promoter site 2 | Forward | CCGCCTGGAGGAAAAATTC |
|  | Reverse | AAGCGACACTGACCTGG |
| *VEGF* promoter | Forward | CAGGAACAAGGGCCTCTGTCT |
|  | Reverse | TGTCCCTCTGACAATGTGCCATC |
| *GAPDH* promoter | Forward | TACTAGCGGTTTTACGGGCG |
|  | Reverse | TCGAACAGGAGGAGCAGAGAGCGA |


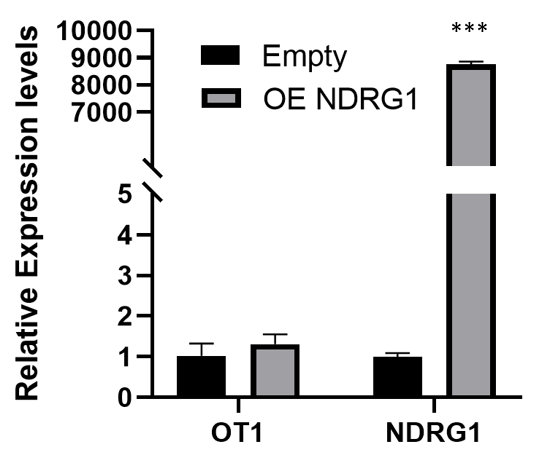


**Figure S1.** Relative expression levels of *NDRG1* and *NDRG1-OT1* in MCF-7 cells overexpressing (OE) *NDRG1-OT1* by quantitative RT-PCR. Loading control: 18S rRNA. ***, *P* < 0.01.

**Fig. 1B: HIF-1α**


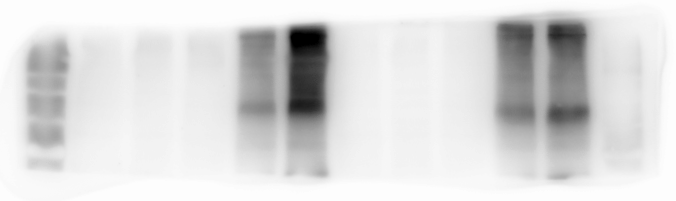


**Fig. 1B: GAPDH**


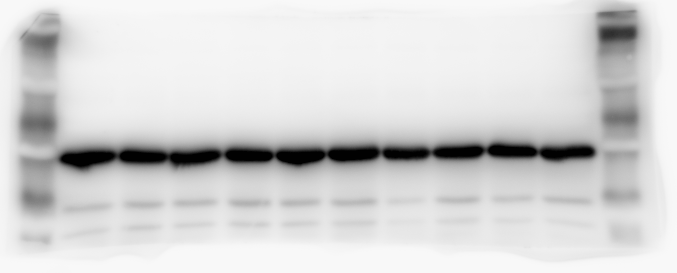


**Fig. 1C: HIF-1α**


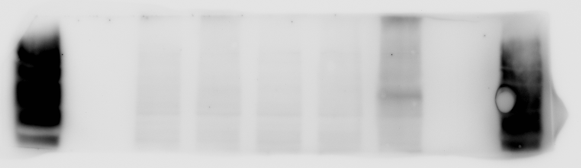


**Fig. 1C: GAPDH**


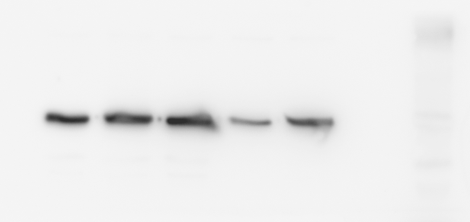


**Fig. 1F: HIF-2α**


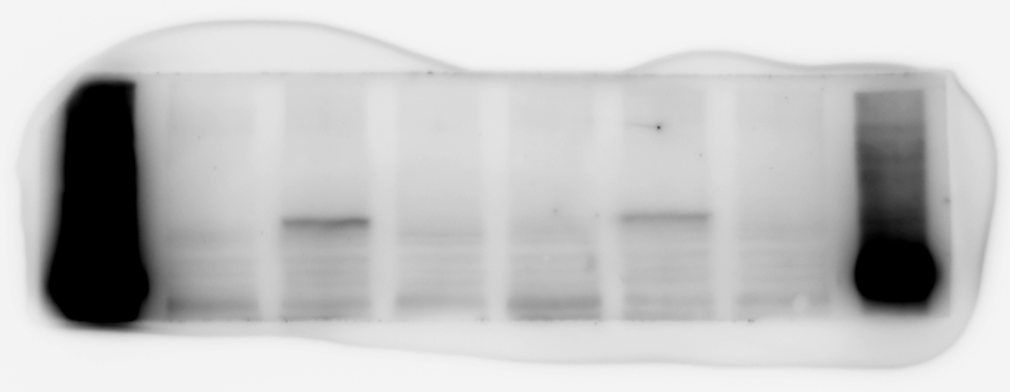


**Fig. 1F: GAPDH**


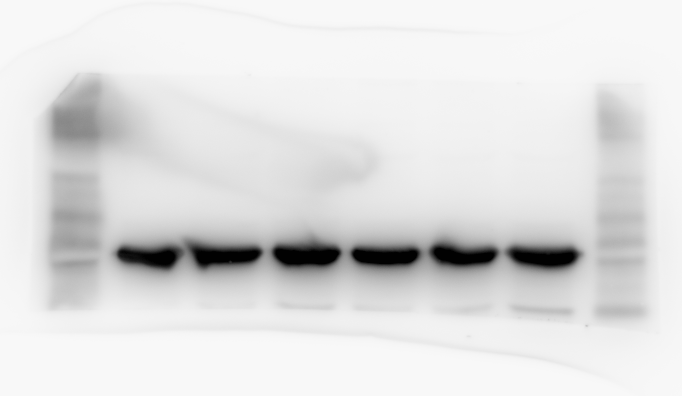


**Fig. 1G: HIF-2α**


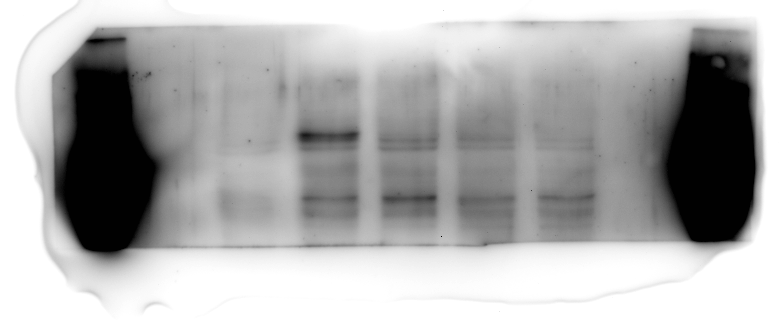


**Fig. 1G: GAPDH**


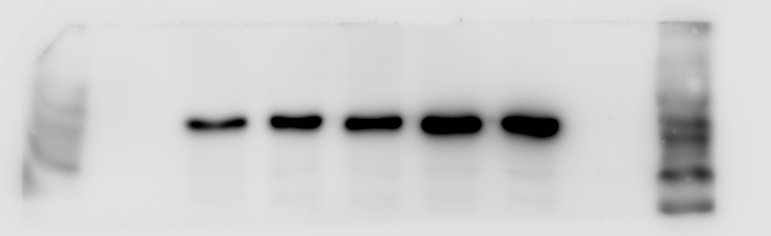


**Fig. 2A: HIF-1α**


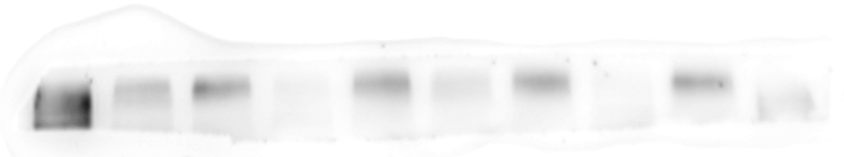


**Fig. 2A: GAPDH**


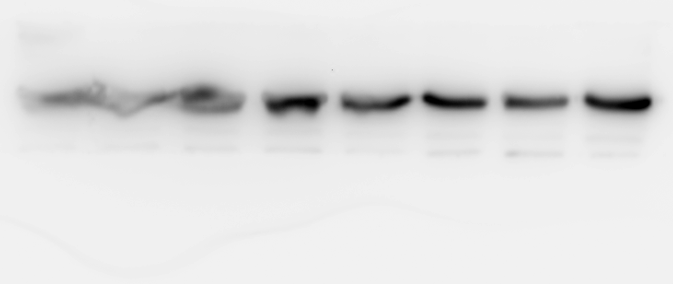


**Fig. 2B: HIF-1α**


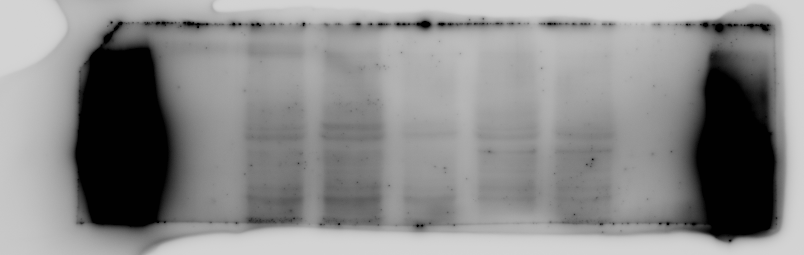


**Fig. 2B: GAPDH**


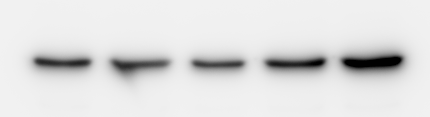


**Fig. 2C: HIF-2α**


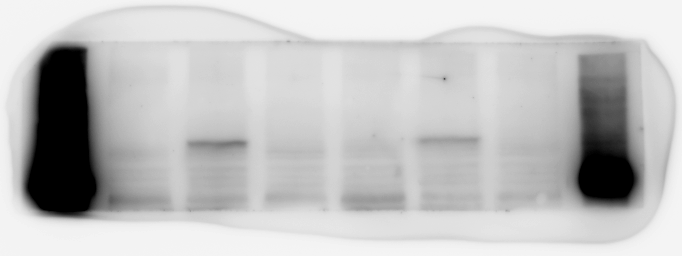


**Fig. 2C: GAPDH**


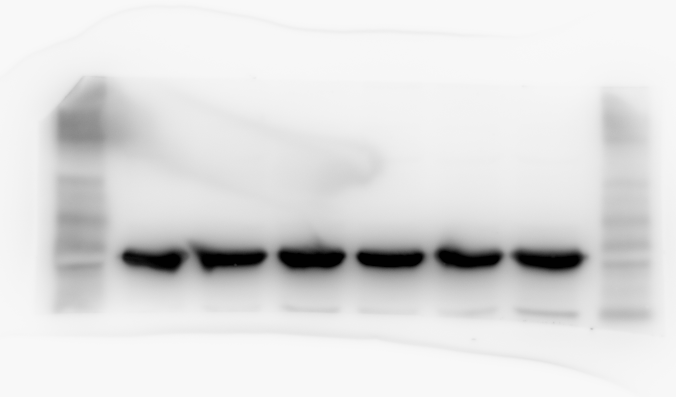


**Fig. 2D: HIF-2α**


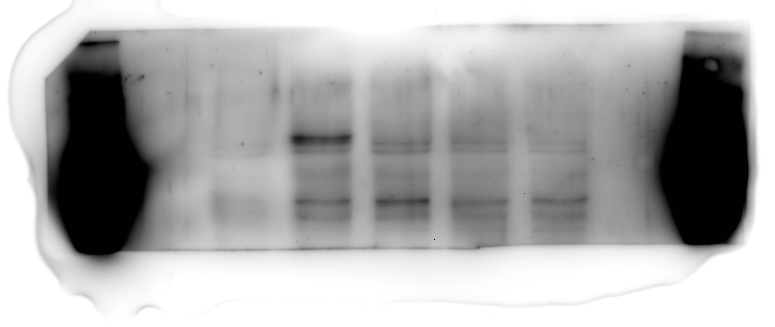


**Fig. 2D: GAPDH**


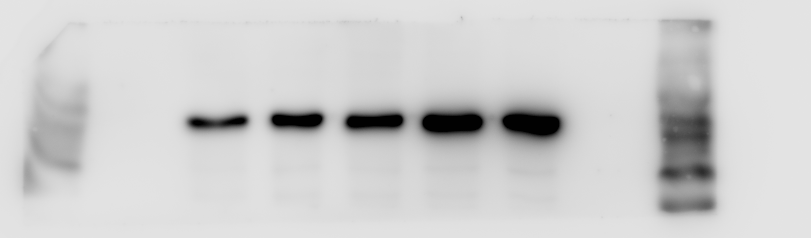


**Fig. 7B: FLAG**


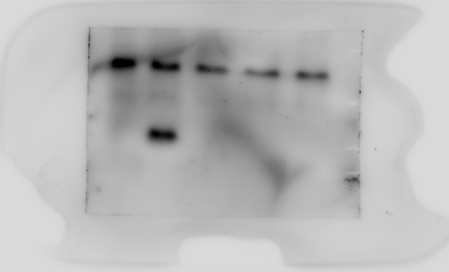


**Fig. 7B: HA**


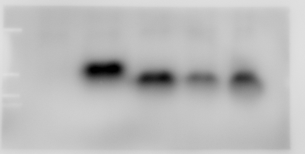


**Fig. 7B: GAPDH**


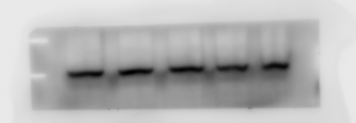

Supplement: Supplementary file 2 — Supplementary materials [file 41419_2022_5253_MOESM2_ESM.docx]
